# Exploring the Impact and Mechanisms of Coffee and Its Active Ingredients on Depression, Anxiety, and Sleep Disorders

**DOI:** 10.3390/nu17193037

**Published:** 2025-09-24

**Authors:** Zijun Shi, Jin Luan, Yating Zhang, Guiping Wang, Can Mei, Linwanyue Chen, Weiji Zhou, Change Xiong, Tao Huang, Jianbo Zhan, Jing Cheng

**Affiliations:** 1 School of Public Health, Wuhan University of Science and Technology, Wuhan 430065, China; 15527761569@163.com (Z.S.); luanjin111@163.com (J.L.); 13469830365@163.com (G.W.); 13235411790@163.com (C.M.); 15357312086@163.com (L.C.); d2051667492@163.com (W.Z.); cherylxiong@wust.edu.cn (C.X.); 2 Hubei Provincial Center for Disease Control and Prevention, Wuhan 430079, China; yating_zh@126.com; 3 Huanggang City Center for Disease Control and Prevention, Huanggang 438000, China; hgcdc2005@163.com

**Keywords:** coffee, active ingredients, depression, anxiety, sleep disorders

## Abstract

Depression, anxiety, and accompanying sleep disorders are common mood disorders worldwide, significantly affecting individuals’ physical and mental well-being as well as their quality of life. Coffee is a widely consumed beverage rich in bioactive compounds, including caffeine, polyphenols, tannins, proteins, and minerals, and studies suggest that coffee and its bioactive constituents demonstrate potential benefits for mental health. However, the specific mechanism by which coffee regulates depression, anxiety and sleep disorders is still unclear, and there is a lack of systematic research in this regard. This study searched four databases (PubMed, Embase, Web of Science, and Google Scholar) to summarize the effects of coffee and its active ingredients on depression, anxiety, and sleep disorders and their mechanisms. Keywords included “coffee”, “active ingredients”, “depression”, “anxiety”, “Sleep disorders “and “mechanisms”. This review summarizes 27 animal studies, 11 clinical studies, and 6 epidemiological studies on this topic. The results showed that moderate caffeine intake may improve mood and cognitive performance, while excessive intake may be associated with anxiety, insomnia, and post-traumatic stress disorder (PTSD). Other compounds, such as polyphenols, may contribute to mental health through antioxidant, anti-inflammatory, and neuroprotective mechanisms. Future research is needed to clarify effective dosages, assess long-term safety, improve coffee processing methods, and explore the development of coffee-based functional foods.

## 1. Introduction

Depression and anxiety often coexist with sleep disturbances [1]. Depression, anxiety and SDs are common mental health problems that significantly interfere with patients’ daily lives, and can lead to other physical health problems [2]. Depression is the leading cause of mental and physical disability, and a major contributor to the global burden of disease worldwide. According to the 2021 WHO, approximately 280 million people (3.8% of the total population) suffered from depression worldwide, and more than 700,000 people died by suicide each year due to depression, with low mood, lack of energy, sadness, insomnia, and the inability to enjoy life [3]. Anxiety is also one of the most common mental health problems worldwide, affecting approximately 25% of the global population, which is characterized by excessive and persistent feelings of fear and worry, that are disproportionate to the actual threats faced, leading to impairment in the patient’s daily life functions [4]. Major depressive disorder (MDD) and anxiety disorders increased by 28% and 26%, respectively, in 2020, with large increases in prevalence among people living in countries severely affected by COVID-19 [5]. SDs are very common, highly treatable conditions that often go untreated, despite their deleterious effects on health and safety. Untreated SDs can reduce alertness and have deleterious effects on cognitive and psychomotor performance [6,7], more than 50% of sleep initiation and maintenance disorders are caused by psychiatric disorders, including addiction [8].

Currently, the treatment and management of depression, anxiety and SDs still face many challenges. In the late 1950s, the first tricyclic drugs (TCAs) were approved for the treatment of depression, mainly imipramine, amitriptyline, clomipramine, desipramine, and doxepin [9]. Other antidepressants include selective 5-HT reuptake inhibitors (SSRIs), fluoxetine, sertraline, paroxetine, and escitalopram [10]; serotonin–norepinephrine (NE) reuptake inhibitors (SNRIs) include milnacipran, duloxetine (DXT), desvenlafaxine succinate (DVS), and venlafaxine [11]. These drugs may cause a range of side effects, including but not limited to weight gain, nausea, sexual dysfunction, and, in certain cases, an increased risk of suicidal tendencies [12]. Canadian clinical treatment guidelines recommend that SSRIs or SNRIs be used as first-line anti-anxiety drug treatment, while benzodiazepines, TCAs, and other drugs are considered second or third-line treatments for panic disorder, generalized anxiety disorder (GAD), and social anxiety disorder (SAD) [13]. However, these treatments are not effective for about a third of patients, who may develop anxiety-related disorders, such as post-traumatic stress disorder (PTSD) and obsessive–compulsive disorder (OCD) [4,14]. Many licensed drugs (including benzodiazepines, daridorexant, suvorexant, and trazodone) are effective for the acute treatment of insomnia, but are poorly tolerated [15]. In recent years, people have been paying increasing attention to natural therapies that utilize natural ingredients from herbs and foods. Such therapies have potential benefits as alternative or adjuvant treatments for mental health problems [16]. For example, in China, Bupleurum has shown some beneficial effects as an adjunctive treatment for post-stroke depression (PSD) [17]. The herb St. John’s wort (hypericum perforatum) may be effective in treating mild to moderate depression [18]. Studies had shown that kava had an anti-anxiety effect on humans [19]. Chinese herbal medicines, such as semen Ziziphus jujube, licorice, and peony had a sedative and hypnotic effect on people with SDs [20].

Coffee, one of the most popular beverages in the world, contains more than a thousand different ingredients, the most well-known of which is caffeine, as well as phenolic acids, diterpenes, trigonelline, tryptophan alkaloids, etc. [21]. Among these identified bioactive ingredients, caffeine can be used as a highly effective stimulant and bronchodilator, while phenolic acids such as chlorogenic acid (CGA), ferulic acid (FA), and caffeic acid (CA) represent the many antioxidant and anti-inflammatory ingredients in coffee. Caffeine remains the most intensively studied compound in coffee, and its effects largely explain why coffee has become such a popular beverage [22]. Coffee has been found to have beneficial effects on human health, such as reduced all-cause and cardiovascular disease-related mortality [23,24], and a lower risk of type 2 diabetes [25,26], Parkinson disease (PD) [27], non-alcoholic fatty liver disease [28], cancer [27,29], and other diseases. Consuming non-toxic doses of caffeine has a variety of positive effects on the brain, including enhanced feelings of well-being, alertness, improved concentration, improved mood, and a reduced risk of depression. However, caffeine may also affect sleep quality in some sensitive individuals and induce anxiety symptoms in some cases [30].

Caffeine is a naturally occurring methylxanthine that acts primarily as a non-selective adenosine A_1_ and A_2A_ receptor (A_1_R and A_2A_R) antagonist and forms functional isomers with dopamine (DA) receptors D1 and D2 in different areas of the brain [31]. Caffeine blocks the binding of A_1_R/A_2A_R to adenosine. This effect was manifested in rat experiments with increased secretion of striatal DA after high dose intake [32]. There is also recent evidence that regular consumption of coffee and caffeinated products can affect resting brain connectivity and influence mood, alertness, and readiness for action [33,34]. The specific performance is that coffee intake reduces the functional connectivity between the posterior default mode network (DMN), and the somatosensory/motor network-prefrontal cortex (PFC) [33]. Furthermore, certain functional networks (involving subcortical areas, visual networks, and cerebellum) showed longer connection durations (signature of longer lifespan) [34].

Despite a long history of coffee consumption, the association between coffee and mood disorders remains unclear. The impact mechanisms, long-term effects and dosage relationships of coffee, and its different bioactive components on mood disorders still need to be further explored. This study aims to systematically evaluate the effects of coffee and its active ingredients on mood disorders (including depression, anxiety, and accompanying sleep disorders) and provide a basis for scientific and rational coffee consumption.

## 2. Methods

### 2.1. Inclusion Criteria

The inclusion criteria are as follows: (1) included studies need to involve coffee drinking or its main active ingredients (such as caffeine, chlorogenic acid, ferulic acid, caffeic acid, etc.); (2) included studies are required to report at least one outcome related to depression, anxiety, or sleep disorders, including but not limited to clinical diagnostic criteria (such as DSM-IV), scale scores (such as depression/anxiety scales), behavioral tests (such as FST, TST, OFT), or biological indicators (such as neurotransmitters, inflammatory factors); and (3) the literature must be written in English and included in the database, with the search time up to 2025.

### 2.2. Exclusion Criteria

The exclusion criteria are (1) articles for which an abstract is not available; (2) non-research articles such as conferences, newsletters, and reviews; (3) duplicate articles; (4) no study outcomes related to depression, anxiety, or sleep disorders are included, only discussing other mental and emotional health issues; (5) coffee or its active ingredients are not mentioned; and (6) the full text is not available.

### 2.3. Search Strategy

**Figure 1:**
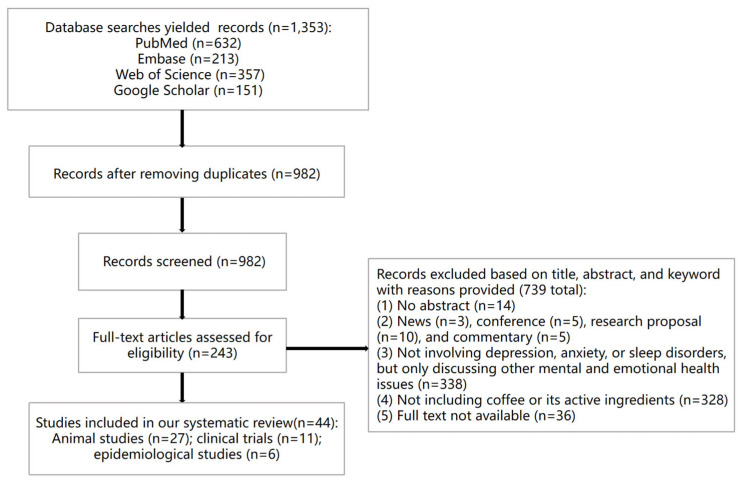
Flowchart of systematic literature search.

We searched electronic databases including PubMed, Web of Science, Embase, and Google Scholar using the keywords “coffee,” “active ingredient,” “depression,” “anxiety,” “sleep disorders,” and “mechanism.” All retrieved study abstracts were read and ranked according to the inclusion and exclusion criteria. The initially included articles were then carefully read and further excluded based on the content of the main articles (Figure 1).

## 3. Associations Between SDs and Depression, Anxiety

SDs are often a sign of a mood disorder diagnosis [35,36]. It is difficult that SDs enter or maintain sleep, which can affect attention and memory in the short term and reduce immunity in the long term, inducing psychological problems such as anxiety and depression. Improving sleep is important in alleviating these symptoms [37,38,39]. The overlap between depression and anxiety is well established; more than 50% of people with depression reported clinically significant anxiety symptoms, and they were more treatment resistant than depressed people without anxiety [40]. SDs also have complex connections with depression. SDs were risk factor for depressive episodes. It is a common physical symptom in MDD and one of the diagnostic criteria for depression based on the Diagnostic and Statistical Manual of Mental Disorders, Fifth Edition (DSM-IV) [41,42]. In addition, insomnia can affect the trajectory of depression, affecting its severity, duration, and recurrence rate [43]. The prevalence of poor sleep quality is high among patients with PD, PTSD, and GAD. Sleep problems can predict the occurrence of anxiety disorders, and many anxiety disorders (such as GAD) already include SDs such as insomnia as key diagnostic criteria [44].

## 4. Effects of Coffee and Its Active Ingredients on Depression

### 4.1. Animal Experimental Study on the Effects of Coffee and Its Active Ingredients on Depression

**Figure 2:**
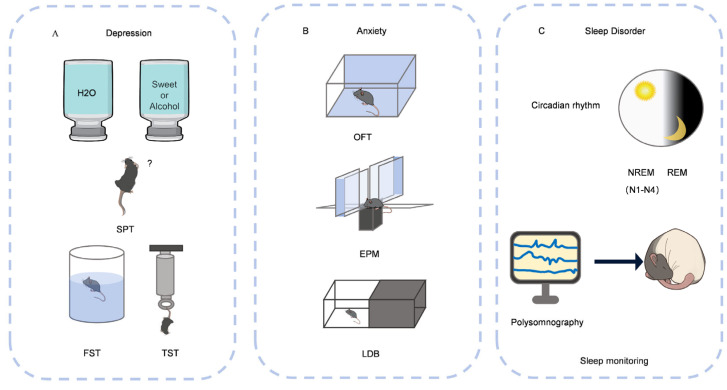
Overview of methods for evaluating depression (**A**), anxiety (**B**), and SDs (**C**) in animal experiments. Notes: The “?” next to the mouse in the picture means the different choices made by the mouse between water and sucrose solution in the SPT.

The sugar preference test (SPT) is used to assess the motivation of mice for a palatable stimulus (1% sucrose solution). Low preference for sucrose (relative to water) is often interpreted as anhedonia in mouse models of depression [45]. The forced swim test (FST) model has been used for decades to test the antidepressant potential of drugs [46]. The tail suspension test (TST) proposed by Steru has a similar depression-inducing mechanism as the FST [47]. The open field test (OFT) is used to determine the depression of model animals based on their activity in unfamiliar open areas [48] (Figure 2A). 

Several ingredients of coffee, such as caffeine [49], CGA [50], FA and CA [51], have shown antidepressant effects in many animal and clinical studies. The amounts [52], type [53], and roast degree [54] of coffee consumed may affect the risk of depression. After consuming coffee and decaffeinated coffee, the abundance of beneficial intestinal flora such as S24-7, Lachnospiraceae, Oscillospira and Akkermansia was restored to varying degrees in rats with chronic paradoxical sleep deprivation model, the levels of pro-inflammatory factors such as interleukin (IL)-6 and tumor necrosis factor (TNF)-α were reduced, and the levels of superoxide dismutase (SOD) and glutathione peroxidase (GPx) were increased. Both coffee and decaffeinated coffee can improve depressive-like behaviors induced by sleep deprivation [53]. Caffeinated coffee reduces indoleamine 2,3-dioxygenase (IDO) activity after exposure to lipopolysaccharide (LPS). Increased uric acid (UA)/3-Hydroxykynurenine (3-HK) and UA/tryptophan-KYN)ratios, the balance of the KYN pathway (KP) in the central nervous system (CNS) shifts from a neurotoxic group to a neuroprotective group, the immobility time in both TST and FST was significantly shortened, which may have an antidepressant effect [55]. Caffeine prevents chronic unpredictable stress (CUS)-induced mood (depression) and memory dysfunction by selectively blocking adenosine A_2A_R mimicry. Treatment of CUS mice with the A_2A_R antagonist (SCH58261) resulted in reduced densities of syntaxin, SNAP-25, and vGluT1, and reduced amplitudes of long-term potentiation (LTP) in the mouse hippocampus. This suggests that synaptic A_2A_R plays a key role in modulating the effects of chronic stress on the brain, which is a candidate target for mitigating the effects of chronic stress on brain function [56]. Caffeine also reduced hippocampal inflammatory response by antagonizing A_2A_R receptors, inhibiting corticosterone (CORT)-induced microglia activation and mitogen-activated protein kinase (MAPK)/extracellular regulated protein kinases (ERK)/nuclear factor kappa-light-chain-enhancer of activated B cells (NF-κB) signaling pathway, thereby restoring neural stem cell proliferation and differentiation, and ameliorating depressive-like behavior in chronic water immersion restraint stress (CWIRS)mice [57].

In addition, caffeine ameliorated chronic mild stress (CMS)-induced depressive-like behavior in mice by targeting the peroxisome proliferators-activated receptor γ coactivator lα (PGC-1α)-kynurenine aminotransferase (KAT) axis in skeletal muscle, inhibiting the neurotoxic branch metabolism of KYN, and promoting its conversion to neuroprotective KYN acid (KYNA). This mechanism is similar to the molecular pathway of exercise antidepressant, and provides a new theoretical basis for caffeine as a potential antidepressant [58]. A study suggested that caffeine’s role in anxiety and depressive behaviors might be gender-specific. Caffeine showed antidepressant effects in the FST assay, with male rats showing primarily increased swimming time, and female rats showing more struggling behavior. This may be related to neurotransmitter systems (such as 5-HT and NE) as well as neural adaptations in the striatum [59]. In addition, there is now a coffee derivative, SY-2476, which has also been shown to have significant anti-depressant effects, improving sugar-water preference, decreasing immobility time, lowering CORT levels, and increasing antioxidant enzyme levels, as well as modulating adenosine A_1_/A_2_A receptor expression in rats [60]. In terms of ingested dose, low doses of caffeine significantly ameliorated LPS-induced depressive-like behavior through anti-inflammatory, antioxidant and up-regulation of brain-derived neurotrophic factor (BDNF) [61]. In terms of coffee intake time, chronic caffeine intake reversed memory deficits but was ineffective against established depressive and anxiety behaviors. Since memory impairment is strongly associated with major depression, caffeine may be better suited as a means of preventing the onset of cognitive decline and depression rather than treating existing symptoms [62,63]. However, the effects of caffeine on depression are not all positive. Studies have shown that caffeine exacerbates depressive-like behaviors in the stress-induced stress (SRS) model of PTSD mice when administered repeatedly (20 and 30 mg/kg). This discrepancy may be due to the use of different mouse models [64]. Caffeine can also improve the subjective well-being of rats by regulating the A_1_R. A study investigating the health of mice showed that caffeine regulated the function of specific brain regions through A_1_R, and that the effects differed by sex: the A_1_R effect was more pronounced in the ventral hippocampus of women, while the effect was more prominent in the dorsolateral striatum of men. Caffeinated coffee improves subjective well-being in both sexes by enhancing A_1_R inhibition in the ventral hippocampus and striatum, which is manifested by increased sociability in men and reduced rank competition and improved self-care in women [65].

CGA pretreatment at the genus level can regulate the relative abundance changes in key bacteria such as Desulfovibrionales, Desulfovibrio, Klebsiella, Burkholderiales and Bifidobacterium. This optimized the gut microbiome structure of rats treated with adrenocorticotropic hormone (ACTH), which may have a positive impact on its anti-depressant effect [50]. When FA is used in combination with drugs such as fluoxetine, paroxetine and sertraline, it can exert an antidepressant effect through 5-HT receptor activity and significantly shorten the immobility time of mice in the TST model, suggesting that the combination of FA and conventional antidepressants may provide a new option for the treatment of depression [66]. CA can reduce the duration of immobility and freezing caused by forced swimming and conditioned fear stress in mice, and its effects are inhibited byα1Aadrenoreceptor (ADRA1A) antagonists. Indirect regulation of the ADRA1A system may be involved in the antidepressant and/or anxiolytic effects of CA [67] (Table 1).

### 4.2. Clinical Study on the Effects of Coffee and Its Active Ingredients on Depression

In clinical trials, the effects of coffee and its components on depressive behaviors have shown inconsistent results. A study of depressed patients treated with intermittent burst theta stimulation (iTBS) or sham treatment of the dorsomedial prefrontal cortex (dmPFC) showed that habitual caffeine intake could enhance the antidepressant effect of iTBS by antagonizing adenosine receptors, and its anti-depressant effect may be related to the enhancement of downstream DA activity [81]. Tse WS [82] asked low-caffeine users to drink caffeinated and decaffeinated coffee and evaluated the effects on social behavior and mood. The results suggest that caffeinated coffee may increase social support behaviors and help improve depressive symptoms. Similarly, Dawkins L et al. conducted a double-blind randomized controlled trial (RCT) in 88 college students and habitual coffee drinkers. The intervention group was divided into two groups: caffeinated and decaffeinated coffee. The results showed that the intake of caffeinated coffee improved attention and psychomotor speed, and had an alleviating effect on depression [83].

However, the results of a double-blind RCT by Loke and Meliska [84] showed that the more caffeine consumed, the higher the alertness, which might have a greater impact on cognitive function, but had no significant effect on depression. A prospective, double-blind, placebo-controlled, crossover study conducted by James and Gregg [85] on 48 rested or sleep-restricted participants showed that caffeine intake had no net effect on the mood of rested subjects, nor did it have a net restorative effect on the depressed mood of sleep-restricted subjects, and even weakened the positive effects of sleep on mood in both conditions (Table 2).

### 4.3. Epidemiological Study on the Effects of Coffee and Its Active Ingredients on Depression

The frequency, amount, and type of coffee intake are all associated with depression. In a prospective study of 9576 Korean adults, those who drank two or more cups of coffee a day had a 32% lower risk of depression than those who did not drink coffee [94]. A prospective study followed 50,739 American women with an average age of 63 for 10 years. The results showed that women who drank 2–3 cups of coffee a day had a 15% lower risk of depression compared to women who drank 1 cup or less a week. Another study also showed that drinking 2–3 cups of coffee a day can reduce the risk of suicide in men and women by about 50% [95,96]. Another cross-sectional study showed that regular coffee and caffeinated coffee intake had a potential protective effect on postpartum women, and consuming more than three cups of caffeinated coffee may reduce the risk of Postpartum Depression (PDD) in women 1–2 years after delivery and in women who are not breastfeeding [97]. A 5-year follow-up study found that the fully adjusted RD (95% CI) for black coffee was −1.7% (−2.6% to −0.7%), suggesting that black coffee might reduce the risk of depression [98] (Table 3).

### 4.4. The Bidirectional Effects of Coffee and Its Active Ingredients on Depression and Future Research Directions

In summary, current studies have shown that coffee and its active ingredients (such as caffeine, CGA) have a bidirectional regulatory effect on depression: moderate intake may improve depressive behavior through anti-inflammatory, antioxidant and neurotransmitter regulation pathways, while high doses or under specific stress conditions may aggravate symptoms. Therefore, coffee cannot be simply equated with a “natural antidepressant.” Epidemiological evidence shows that drinking 2–3 cups of coffee per day is significantly associated with a reduced risk of depression, but clinical results are inconsistent, which may be related to the intake dose, individual metabolic differences and coffee type (black coffee, decaffeinated coffee).

Future research may focus on (1) the synergistic antidepressant mechanism and dose–effect relationship of different components in coffee or (2) the effects of long-term coffee intake on special populations (such as postpartum women and individuals with chronic stress). It is also important to clarify the impact of coffee processing methods (roasting degree) on the retention of active ingredients and antidepressant efficacy.

## 5. Effects of Coffee and Its Active Ingredients on Anxiety

### 5.1. Animal Experimental Study on the Effects of Coffee and Its Active Ingredients on Anxiety

OFT and elevated plus maze test (EPM) are important behavioral methods for assessing animal anxiety level and motor function, and are widely used in neuro-psychopharmacology research [100]. CGA, caffeine, and coffee all improved brain enzyme activity disorders in streptozotocin-induced diabetic rats, prevented the increase in synaptic acetylcholinesterase (AchE) activity in the cerebral cortex, and reduced OS levels by reducing thiobarbituric acid reactive substances (TBARS) levels, thereby improving memory and significantly reducing anxiety [70]. Both acute and chronic low-dose caffeine reverses stress-induced anxiety-like behavior and cognitive deficits, and it is more suitable as an intervention to prevent stress-related cognitive decline and anxiety [68]. Yet another new study suggested that caffeine reduced anxiety behaviors in the short term, but this anxiety-reducing effect faded with long-term intake, though it did enhance memory in the chronic phase [69]. Other study had shown that caffeine (20 and 30 mg/kg, i.p.) injected intra-peritoneally exacerbated anxiety-like behavior induced by the stress model (SRS), as evidenced by mice exploring the open area less in the elevated plus maze (days 14 to 32). Therefore, the effect of caffeine intake on anxiety is not constant, but presents a complex and inconsistent result (Figure 2B).

Isochlorogenic acid (ICCA) improves the function of the cholinergic system and enhances antioxidant and anti-inflammatory capabilities by activating the BDNF/nuclear factor-erythroid 2 related factor 2 (Nrf2)/GPx4 pathway, thereby alleviating lead-induced neuroinflammation, ferroptosis, and OS in mice and significantly reducing anxiety-like behaviors [71]. The researchers used the light/dark box test (LDB), EPM and free exploration test (FET) to explore the anxiolytic effect of CGA in a mouse anxiety model. In LDB, CGA can increase the number of transitions from the dark box to the light box, the cumulative time in the light box, and the motor ability of mice, showing an anti-anxiety effect. In the EPM experiment, the cumulative time and total number of visits in the open arm of the CGA treatment group (20 mg/kg) increased significantly. In FET, mice given CGA (20 mg/kg) and diazepam (1 mg/kg) showed more movement and exploration behavior in unfamiliar environments than the control group. These results confirmed the anxiolytic effect of CGA. Its mechanism of action may be that the pharmacological characteristics of CGA at 20 mg/kg are similar to those of the benzodiazepine diazepam, and it may produce anxiolytic effects by activating benzodiazepine receptors [72]. The combination of CGA and Syringaresinol-di-O-glucoside (SYG) may induce anxiolytic behavior and modulate autonomic nervous system regulation, activate hippocampal BDNF signaling, and attenuate the inhibitory effect on stress-induced peripheral nervous system (PNS) activity [73].

FA restored the 20-carboxy-leukotriene B4 in LPS-induced mice to normal levels, and the ratio of Firmicutes/Bacteroidetes also increased to normal levels. It can also increase the level of 5-hydroxyltryptophan (5-HTP), the precursor of 5-HT, to prevent anxiety [75]. When rats were fed a high-fat diet and exposed to chronic stress, CA modulated the β-catenin/glycogen synthase kinase-3β (GSK-3β) pathway, thereby affecting the function of nerve cells, neurotransmitter levels, neuroplasticity and other related physiological processes, and might have a neuroprotective effect on cognitive changes and anxiety-like behaviors [74] (Table 1).

### 5.2. Clinical Study on the Effects of Coffee and Its Active Ingredients on Anxiety

A prospective, double-blind, placebo-controlled study of 56 women showed that moderate caffeine intake (low to moderate doses) slightly reduced anxiety in postmenopausal women with underlying overactive bladder (OAB), but had no significant effect on depressive symptoms [86]. In an open-label experimental study by Kimura T et al. [88], 20 patients with frontotemporal lobar degeneration or Lewy body dementia took FA and angelica extract (Feru-guard) 3.0 g daily for 4 weeks. Among them, 19 subjects had a significant decrease in the overall scores of the neuropsychiatric scale and its subscales (including “agitation/aggression”, “anxiety”, etc.), indicating that Feru-guard might have an alleviating effect on anxiety. Quinlan P et al. [87] monitored the effects of caffeine withdrawal on physiological and emotional indicators by having subjects drink hot tea, coffee or water with or without caffeine. The results showed that caffeine can improve mood and reduce anxiety. A RCT of 62 normal adults showed that subjects who consumed caffeine on a daily basis experienced withdrawal symptoms such as depression, anxiety, fatigue and headache after stopping caffeine intake, which might indicate from another perspective that caffeine intake could reduce anxiety reactions [101] (Table 2).

By contrast, a study by Chait LD et al. [102] had smokers take a placebo, different doses of caffeine, or d-amphetamine before smoking and evaluated the effects on their subjective responses. The results showed that caffeine could cause negative subjective reactions, such as nervousness, anxiety and irritability. A double-blind RCT by Nardi AE et al. [103] also showed that patients with PD were more likely to have panic attacks induced by caffeine intake than healthy people, which suggested that caffeine might aggravate anxiety symptoms.

### 5.3. Epidemiological Study on the Effects of Coffee and Its Active Ingredients on Anxiety

A large prospective cohort study showed that people who drank 2–3 cups of coffee per day had the lowest risk of anxiety. In addition, coffee subtypes including 2–3 cups of ground coffee, milk coffee, and unsweetened coffee were associated with a reduced risk of anxiety [52]. Another cohort study of 180,000 participants aged ≥ 60 years showed that drinking ≥ 1 cup of coffee per day was associated with a lower risk of depression and anxiety disorders [99]. The current results indicate that several proteins (including inflammatory and other proteins) from neural-related tissues (brain, cerebrospinal fluid (CSF) and plasma) interact with coffee and affect anxiety, such as brain protein c-Jun, CSF protein Fas, IL-6, IL-1sRI, and MIP-5. The interaction between CSF protein IL-6 and coffee intake was negatively associated with anxiety, and the interaction between coffee intake and brain protein c-Jun was positively associated with anxiety [104].

However, a cohort study of 941 overweight European adults with subsyndromal depression did not find a relationship between coffee consumption and levels of depression and anxiety [105]. In a recent systematic review, caffeine equivalent to five cups of coffee induced anxiety in both patients with PD and healthy controls [106]. These different conclusions may be attributed to differences in the subjects’ age, gender, physical condition, and different methods of assessing the severity of depression and anxiety (Table 3).

### 5.4. Bidirectional Regulation of Anxiety by Coffee and Its Active Ingredients Intake and Future Research Directions

Existing animal experiments and clinical studies have shown that coffee and its active ingredients have a bidirectional effect on anxiety: low-dose or short-term intake may relieve anxiety by regulating neurotransmitters and antioxidant pathways, while high-dose or long-term intake may aggravate anxiety symptoms, especially in susceptible populations (such as Parkinson’s disease patients). Epidemiological evidence suggests that moderate coffee consumption (2–3 cups per day) is associated with a reduced risk of anxiety, but the results are influenced by individual differences (metabolic capacity, genotype) and coffee type.

Future studies could further focus on the long-term effects of coffee consumption on anxiety in special populations, such as patients with neurodegenerative diseases. Additionally, the association between specific types of coffee (decaffeinated coffee and milk coffee) and anxiety is also worth exploring.

## 6. Effects of Coffee and Its Active Ingredients on SDs

### 6.1. Animal Experimental Study on the Effects of Coffee and Its Active Ingredients on SDs

Habitual caffeine drinkers may have a potential tolerance to caffeine, which may mitigate its deleterious effects on subsequent sleep. Regular caffeine intake will cause the CNS to be continuously exposed to the caffeine environment. When rodents are repeatedly exposed to caffeine, the adenosine system will produce an adaptive response (increased brain adenosine concentration and upregulation of brain adenosine receptors) to cope with caffeine and its metabolites. SDs may be alleviated with repeated caffeine intake [107]. Caffeine disrupts physiological sleep and enhances wakefulness by inhibiting A_2A_R [76,108]. Caffeine can play a dual protective role by acting on the A_2A_R of brain vascular endothelial cells: structurally maintaining the integrity of the blood–brain barrier to prevent harmful substances from penetrating into the brain parenchyma; functionally optimizing cerebral blood flow and vascular microenvironment, indirectly supporting the survival and function of neurons [109]. In addition, the neuroprotective effects of caffeine may be related to increased blood flow during sleep, which also helps clear metabolic waste from the brain [77].

Activation of orexin neurons by caffeine through antagonism of adenosine receptors (A_1_ and A_2_A) increases arousal levels and sympathetic nerve activity in sleep-deprived mice, while orexin receptor antagonists (OXRs) antagonists block the pro-arousal effects of caffeine, suggesting that the orexin system serves as an important target of caffeine-regulated sleep, providing a potential direction of research for the prevention of caffeine-induced arousal [79]. Both CGA and its metabolite CA can cause a significant increase in sleep latency in rats. The α1-adrenergic receptor (α1-AR) system may contribute to the awakening effect of CA, but neither affects the overall sleep–wake cycle [78] (Table 1).

### 6.2. Clinical Study on the Effects of Coffee and Its Active Ingredients on SDs

A crossover study conducted on 16 adult men showed that after drinking a beverage containing 300 mg of CGA for 2 consecutive weeks, the CGA group felt less tired when waking up and their sleep quality was significantly improved [89]. In another study, 9 healthy men and women took a drink containing 600 mg of CGA for 5 days. Park et al. found that CGA shortened sleep latency and enhanced parasympathetic nerve activity compared to the control group without adversely affecting sleep quality [90].

A double-blind crossover study in which 12 young people and 12 middle-aged people took 200 mg of caffeine or a placebo showed that caffeine prolonged sleep latency, reduced sleep efficiency and duration, and reduced low-frequency electroencephalogram (EEG) activity and increased high-frequency activity [110]. Marcus GM et al. [111] conducted a randomized case-crossover trial in 100 adults, in which the subjects drank caffeinated coffee or avoided caffeine for 14 days. The results showed that consuming caffeinated coffee significantly reduced the amount of sleep per night (approximately 36 min). Burke TM et al. [91] found that consuming about 200 mg of caffeine 3 h before bedtime can cause a phase delay of about 40 min in the body’s melatonin secretion rhythm, thereby affecting the CR. At the same time, genetic experiments on osteosarcoma cells further confirmed that caffeine could prolong the cycle of circadian clock gene expression in a dose-dependent manner. Ramos-Campo DJ et al. [92] asked 15 middle-and long-distance runners to take 6 mg/kg caffeine or placebo 1 h before an 800-m running test in the evening and evaluated their athletic performance and sleep quality. The results showed that caffeine did not significantly improve running performance, but significantly reduced sleep efficiency, increased the number of awakenings, and impaired subjective sleep quality. However, a study of 20 habitual caffeine consumers who were given either 150 mg of caffeine three times a day or a placebo for 10 consecutive days showed that daily caffeine intake did not significantly affect total sleep time or subjective sleep quality [93] (Table 2).

### 6.3. Potential Research Directions for Caffeine’s Effects on Sleep

Existing research shows that coffee and its active ingredients have different effects on sleep: caffeine may affect the sleep–wake cycle by regulating adenosine receptors and circadian clock genes, which may prolong the time to fall asleep and reduce sleep efficiency in the short term, but long-term intake may alleviate the negative effects through adaptive mechanisms; CGA shows the potential to improve sleep quality, such as shortening the time to fall asleep and reducing fatigue. Individual responses vary significantly, which may be related to the dose, time of intake and metabolic characteristics.

Future research may further explore (1) the combined effects of the synergistic or antagonistic effects of different components in coffee on sleep; (2) personalized intake recommendations based on individual metabolic differences (genotype, caffeine metabolism rate); and (3) optimizing the coffee intake time window to balance daytime alertness and nighttime sleep quality. In addition, coffee usage strategies for special groups (such as athletes and shift workers) are also worthy of further discussion.

## 7. Potential Mechanisms of Action of the Main Active Ingredients in Coffee on Depression, Anxiety, and Sleep Disorders

**Figure 3:**
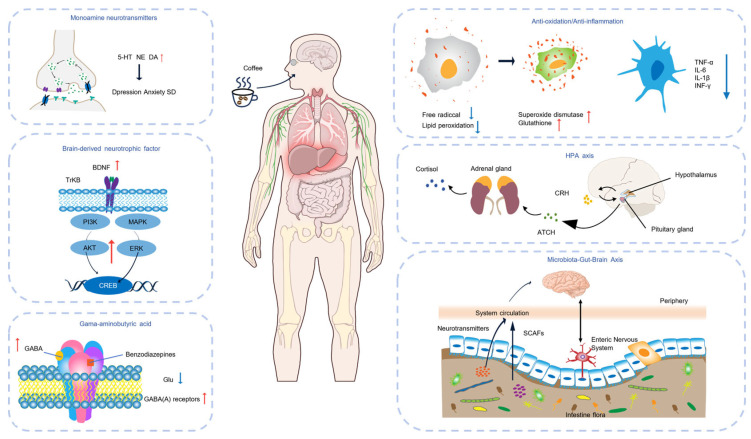
Potential mechanisms of action of the main active ingredients in coffee on depression, anxiety, and sleep disorders. Notes: Blue “↓” represents downregulation, red “↑” represents upregulation, black arrows represent the path and direction of the mechanism in the figure.

Coffee beverages are complex chemical mixtures extracted from coffee beans. Their bioactive components may produce combined effects in the body through pharmacokinetic interactions (such as absorption, distribution, metabolism and excretion) as well as synergistic or antagonistic pharmacodynamic effects. Therefore, the overall physiological effects of coffee may differ significantly from those observed when studying any of its components in isolation. This study summarizes the potential mechanisms of action of these main active ingredients that have been reported. The effects of coffee beverages on depression, anxiety and sleep disorders may be the result of the interaction of these complex mechanisms (Figure 3).

### 7.1. Caffeine and Adenosine Receptors

Adenosine is an important nucleoside molecule that plays a key regulatory role in various physiological processes, such as energy metabolism, neurotransmission and immune regulation by binding to four subtypes of adenosine receptors, namely A_1_, A_2A_, A_2B_ and A_3_ [112,113,114]. In the hippocampus, A_1_R mainly inhibits the release of neurotransmitters before the synapse, reduces synaptic transmission and neuronal excitation, and plays a neuroprotective role [115,116]. It enhances postsynaptic neural plasticity by regulating n-methyl-d-aspartate (NMDA) receptor-dependent LTP. A_2A_ receptor antagonists (such as caffeine) may block A_2A_ receptor-mediated NMDA receptor-dependent LTP and affect synaptic plasticity [117]. A_2A_R significantly promotes glutamate release when A_1_R activity is partially inhibited (such as in the presence of N^6^-Cyclopentyladenosine). It also enhances synaptic transmission by directly regulating glutamate release through the protein kinase C (PKC) pathway, rather than relying on changes in cyclic adenosine monophosphate (cAMP) levels [118]. Stress-induced changes in the adenosine neuromodulatory system are initially an adaptive response to homeostasis, but under chronic stress conditions, persistent changes in the adenosine neuromodulatory system can lead to synaptic dysfunction, which is considered to be the root cause of depressive-like behavior [109,119,120]. Aberrantly increased A_2A_R signaling in the lateral septal (LS)nucleus is a key upstream regulator of stress-induced depressive-like behavior. Based on this mechanism, A_2A_R antagonists may become an effective antidepressant [120].

Caffeine’s effects on the brain and CNS are primarily through antagonism of adenosine A_1_R and A_2A_R, which blocks the normal inhibitory function of adenosine itself [56,121,122]. Within the safe dosage range, the mechanism of action of caffeine on synaptic function and neural plasticity in the cerebellar hippocampus mainly depend on its antagonistic effect on adenosine receptors. Among them, adenosine A_1_R mediates the regulation of caffeine on synaptic signal transmission, while adenosine A_2A_R participates in the caffeine-induced synaptic LTP process through functional regulation [123,124]. Caffeine modulates synaptic function by blocking A_2A_R, thereby improving mood-related behaviors [56]. The ADORA2A gene is a candidate gene for anxiety, and caffeine may induce more pronounced anxiety responses in people with specific A_2A_R gene polymorphisms [125]. The rs2298383 single nucleotide polymorphism (SNP) may affect the function of A2A receptor by regulating ADORA2A gene expression. The rs 2298383-TT genotype is associated with a reduced risk of depression, indicating the important role of ADORA2A gene expression in emotion regulation. It is also related to the protective effect of sleep dysfunction [126].

### 7.2. Monoamine Neurotransmitter Regulation

#### 7.2.1. Effects of Caffeine on DA

Caffeine can stimulate the release of 5-HT in the limbic system and promote the release of DA in the PFC, producing effects similar to those of anti-depression drugs [127,128]. However, the regulatory mechanism of caffeine on DA release is still controversial. Although caffeine may enhance the activity of DA D1 receptors by interfering with the action of adenosine A_1_R [129], the coexistence of adenosine A_2A_R in dopaminergic nerve endings may antagonize this effect, and blockade of A_2A_R will reduce DA release [130,131]. Therefore, the effect of caffeine on DA release is not simply mediated by the A_1_R, and the final result may depend on the dynamic balance of dual regulation of A_1_R and A_2A_R. There are also studies showing that acute exercise-activating doses of caffeine produce a weak antagonistic effect on A_2A_R, which may not be sufficient to offset caffeine’s antagonistic effect on presynaptic A_1_R [132]. This may indirectly lead to an increase in DA and glutamate release.

#### 7.2.2. Effects of Coffee and Its Active Ingredients on Monoamine Mechanisms in Depression

Initial evidence supporting the “monoamine hypothesis of depression” was that monoamine oxidase inhibitors and tricyclic antidepressants can improve depressive symptoms by enhancing the activity of 5-HT and NE [133]. The monoamine deficiency hypothesis may not be prevalent in depressed patients, suggesting that other pathways and neurotransmitters are involved in depression [134]. Caffeine has an inhibitory effect on endogenous adenosine, which reduces the release of many neurotransmitters in the CNS, including 5-HT, thereby indirectly protecting the release of 5-HT [135]. One study showed that dietary caffeine administration in rats resulted in increased CNS concentrations of 5-HT, 5-hydroxyindoleacetic acid (a breakdown product of 5-HT),and trypophan (a starting material for the production of 5-HT) [136]. The active ingredients in coffee may also work by regulating monoamine metabolism. Coffee ingredients such as trigonelline, n-methylpyridine, CGA, catechol, and pyrogallol have been shown to increase calcium signaling and DA release, thereby exerting antidepressant effects [49]. Caffeine, FA, and CA inhibit the reuptake of 5-HT, increase the content of synaptic cleft, and affect the occurrence of depression. Chronic caffeine intake (8 mg/kg/d) can also affect the 5-HT level in the hippo-campus [51]. Monoamine oxidase-A (MAO-A), an X-linked mitochondrial enzyme, degrades key neurotransmitters such as 5-HT and DA [137]. CGA in Robusta coffee inhibits MAO-A activity, preventing 5-HT oxidative deamination and thereby elevating synaptic 5-HT levels, this suggests that coffee may be a potential antidepressant [54].

#### 7.2.3. The Effect of Caffeine on Anxiety in the Threat–Fear Circuit

Typically, the neural circuit most closely associated with anxiety disorders is the threat–fear circuit [138]. Caffeine can change the human brain’s response pattern to social threat cues, inducing activation of the threat-related midbrain-periaqueductal gray (PAG) area, which may be linked to the threat-related activity involved in panic attacks and PD [106], and eliminating threat-related activation of the medial prefrontal cortex (mPFC) [139]. Furthermore, the effects of caffeine on the magnitude of threat-related amygdala activation were related to levels of dietary caffeine intake [139]. Deviations in threat prediction may cause the defense system to be over-engaged, leading to anxiety [140].

Caffeine increases threat-related activity in the PAG, which may be linked to the threat-related activity involved in panic attacks and PD [106]. Caffeine modulates hippocampal synaptic plasticity to enhance working and reference memory by blocking adenosine A_2A_R. A_2A_R are mainly enriched in the presynaptic terminals of the amygdala, which participate in the formation and consolidation of fear memory by controlling synaptic plasticity, and the downregulation will damage fear memory. Therefore, caffeine may reverse abnormal synaptic plasticity by antagonizing the A_2A_R, thereby reducing pathological fear memory (such as PTSD) [141].

#### 7.2.4. Mechanisms of Caffeine’s Effects on Sleep and Circadian Rhythm (CR)

Coffee can inhibit adenosine in melatonin secretion, and its secretion level shows specific CR changes. Therefore, caffeine intake will change the CR of melatonin and cause SDs [142]. There is evidence that caffeine intake mainly delays the human melatonin rhythm by antagonizing A_1_R, increasing cAMP levels, affecting Per1/Per2 transcription through the cAMP-Protein kinases-CREB signaling pathway [91]. Since adenosine receptors are present in the SCN, which acts as a central biological clock and controls the pineal gland through polysynaptic connections, caffeine may cause desynchronization of various circadian systems driven by the SCN. Flavonoids, which are abundant in coffee, have also been shown to inhibit the activity of aryl alkylamine N-acetyltransferase (AANAT) and reduce serum melatonin levels in rats at night [143]. Adenosine acts through the A_1_R/A_2A_R signaling pathway, activating the Ca^2+^-ERK-AP-1 and CREB/CREB-regulated transcription coactivator 1 (CRTC1)-cAMP response element (CRE) pathways, thereby regulating the expression of the clock genes Per1 and Per2. As an adenosine A_1_R/A_2A_R antagonist, caffeine can restore the sensitivity of SCN to light through the adenosine receptor pathway and shorten the adjustment time of CR [80]. Adenosine promotes sleep, which inhibits neuronal activity by activating the A1R in the visual cortex, thereby inducing and maintaining non-rapid eye movement sleep (NREM) and rapid eye movement sleep (REM) sleep. As a non-selective adenosine receptor antagonist, caffeine may interfere with this signaling pathway, thereby weakening or completely blocking the sleep aid effect brought by light. Therefore, avoiding caffeine intake may be a key factor in ensuring efficacy in 40 Hz light treatment of SDs based on adenosine mechanism [144].

Caffeine regulates the release and metabolism of monoamine neurotransmitters (DA and 5-HT) by antagonizing adenosine receptors (A_1_R/A_2A_R), exerting a complex dual regulatory effect on emotional expression. Therefore, targeted emotion intervention strategies based on receptor subtype specificity (A_1_R/A_2A_R) can be further studied in the future. In addition, the impact of individual metabolic differences (genetic polymorphisms) on the neuromodulatory effects of caffeine is also worth exploring.

### 7.3. Non-Monoamine Neurotransmitter Regulation

#### 7.3.1. Caffeine Regulates Neural Function Through BDNF Signaling Pathway

BDNF is a member of a unique family of neurotrophic growth factors [145]. BDNF plays multiple key roles in brain development and function. It not only promotes the growth, maintenance and protection of neurons, but also participates in the regulation of nerve regeneration and synaptic plasticity. Studies have shown that BDNF plays a key regulatory role in LTP of excitatory synapses in the hippocampus and other brain regions, and plays an important mediating role in the formation of learning and memory [146,147,148,149]. BDNF binds with high affinity to the tropomyosin-related kinase B (TrkB) receptor, thereby activating signaling pathways that regulate many synaptic processes [150]. After BDNF binds to the TrkB receptor, it activates two core signaling pathways, phosphatidy-linositol 3-kinase/mechanistic target of rapamycin (PI3K/mTOR) and mitogenactivated protein kinase (MAPK)/ERK, by phosphorylating Srchomology domain 2 and phospholipase C-γ (PLC-γ) [151,152].

BDNF levels can signal changes in neuronal activity and can also participate in homeostatic plasticity processes, mediating synaptic scaling [153,154,155]. The timing of BDNF release modifies its effects on homeostatic plasticity. Acute administration of BDNF in the nucleus accumbens (NAc) increases the expression of α-amino-3-hydroxy-5-methyl-4-isoxazolepropionic acid receptor (AMPAR) subunits, whereas chronic administration leads to downregulation of their expression [154]. TrKB, as a BDNF receptor, not only senses cholesterol through its transmembrane domain and regulates its synaptic effects, but also directly binds to typical and rapid antidepressants, thereby promoting their synaptic localization and BDNF-dependent activation, ultimately mediating neuronal plasticity and antidepressant responses [156].

Caffeine antagonizes presynaptic adenosine A_1_R and promotes the mobilization of Ca^2+^ from ryanodine-sensitive intracellular stores. The increase in intracellular Ca^2+^ concentration may trigger the secretion of BDNF near the synaptic cleft, which in turn activates the TrkB receptor and phosphorylates insulin receptor substrate 2 (IRS2) at the corresponding site, thereby recruiting the PI3K/protein kinases B (Akt/PKB) signaling pathway. Furthermore, caffeine induced an NMDA receptor-independent LTP, termed CAFLTP (caffeine-induced LTP), in the CA1 region of the hippocampus by promoting Ca^2+^-dependent BDNF secretion. This process is dependent on TrkB-mediated signaling, contributes to CAFLTP expression, and alleviates depressive symptoms [157,158]. Caffeine can reverse the stress-induced decrease in basal phosphorylated calcium calmodulin kinase I (P-CaMKII), total CaMKII and BDNF protein levels in the CA1 region of the hippocampus and restore them to normal levels. Restoration of these signaling molecules may be responsible for the beneficial effects of caffeine on LTP under stressful conditions [159]. BDNF is also believed to be involved in the pathogenesis of anxiety disorders [160]. The role of BDNF in regulating synaptic plasticity may underlie its effects on conditioned fear responses and anxiety regulation [161,162,163]. A_2A_R deficiency in the hippocampus may impair specific fear conditioning and reduce anxious behavior by interfering with the BDNF-TrkB pathway [163]. Caffeine may be involved in the regulation of BDNF levels in the frontal cortex by antagonizing A_2A_R, and in the caudate putamen, the effect of caffeine on BDNF expression depends on the individual’s level of anxiety-like behavior, that is, BDNF expression is increased in high anxiety-like behaviors, but not in low anxiety-like behaviors [164].

#### 7.3.2. Caffeine Regulates Neuronal Function via Them Gamma-Aminobutyric Acid (GABA) Pathway

In the human body, GABA mainly acts as a neurotransmitter in the CNS and has the function of inhibiting nerve excitability. In addition, it is also involved in a variety of physiological processes, such as regulating sleep, blood pressure, and anxiety [165]. Cholecystokinin (CCK)-GABA neurons (by injecting AAV-mDlx-DIO-hM4Di-mCherry virus into CCK-Cre mice) can participate in regulating depression-like and anxiety-like behaviors [166]. Traditionally, antidepressant drugs that enhance the function of 5-HT and NE achieve their antidepressant effects by enhancing the function of GABAergic interneurons [167]. The GABAergic output of the anteroventral nucleus of the bed nucleus of the stria terminalis (avBNST) affects the activity of DA neurons in the ventral tegmental area (VTA) and serotonergic neurons in the dorsal raphe nucleus (DRN). Blocking the presynaptic GABAB receptors of the avBNST can inhibit its GABAergic neurons, reduce the inhibitory output to the VTA and DRN, increase the levels of DA and 5-HT in the basal lateral amygdala (BLA), and produce anxiolytic effects [168]. GABAB receptor antagonists are considered effective antidepressants [169]. Caffeine’s enhancing effect on GABA release is mediated by blocking adenosine A_1_R [170]. Caffeine can temporarily reduce inhibitory postsynaptic currents of GABAergic pathways in hippocampal CA1 pyramidal cells, mainly mediated by the phosphodies-terase pathway. When inhibitory GABAergic signaling is attenuated, it may lead to increased activity of the dopaminergic system [171,172]. Under conditions of caffeine tolerance, the reduction in GABAergic activity can be restored to normal, suggesting that caffeine dose and duration of treatment may play a role in modulating GABAergic signaling [173]. Caffeine-induced protection of GABAergic and Glu neurons mediates the improvement of neurobehavioral responses [170,174].

In summary, caffeine may participate in the regulation of emotional state by regulating the BDNF signaling pathway and GABA pathway. Among them, BDNF’s key role in synaptic plasticity, learning and memory, and emotion regulation makes it an important target for caffeine to affect neural function. While the GABA system is directly related to behavioral manifestations such as anxiety, sleep, and depression. The effects of caffeine on these pathways may be dose-dependent and individual-varying. In the future, further attention can be paid to the specific regulatory patterns of caffeine intake at different doses and durations on the BDNF signaling pathway and the GABA pathway, especially the changes in neural function under stress, anxiety-like behavior and depression.

### 7.4. Oxidative Stress (OS) and Inflammation

#### 7.4.1. The Mechanism of Action of Coffee and Its Active Ingredients on Emotions Under OS

OS is caused by a disturbance in the normal balance between the production of free radicals (especially reactive oxygen species (ROS)) and antioxidant defenses [175]. Increased reactive oxygen species can modulate hypothalamic–pituitary–adrenal (HPA) axis feedback, inducing its over activation and altering GABA and 5-HT energy transmission [176,177,178]. Activation of the HPA axis in turn promotes the release of glucocorticoids (GCs), which can affect the cell’s redox system through their receptors. Activated glucocorticoid receptors (GRs)can cause increased mitochondrial membrane potential, imbalance of calcium homeostasis, and enhanced mitochondrial oxidation, which further increases the generation of ROS such as superoxide (O_2_^−^), hydrogen peroxide (H_2_O_2_), and hydroxyl radicals (·OH). This cycle ultimately leads to increased OS and may induce cellular oxidative damage [179,180,181]. The hypoxic process disrupts neurohormonal homeostasis in the brain and increases the likelihood of depression by promoting inflammation, apoptosis, 5-HT pathway dysregulation, and mitochondrial OS [181,182]. OS also plays an important role in the pathophysiology of anxiety. The CNS (such as hippocampus, cortex) and peripheral immune cells (such as white blood cells) in anxiety disorders show enhanced OS [183]. Intake of antioxidant supplements such as magnesium, zinc, selenium and Coenzyme Q10 is significantly associated with a decrease in depression and anxiety status, especially with significant effects on improving depression symptoms [184].

Caffeine is a well-proven antioxidant with an activity comparable to glutathione. Caffeine and its metabolites theobromine and xanthine can prevent the production of free radicals such as (·OH), peroxyl radicals (ROO·) and singlet oxygen (^1^O_2_), thereby reducing lipid peroxidation in vitro [185,186,187]. After OS caused by hydroxyl and ROO·, the protein oxidation, lipid peroxidation and ROS levels in the synaptic membrane increased significantly. FA can greatly alleviate these oxidative damage and prevent the conformational changes in synaptic membrane proteins caused by free radicals [188]. CA can remove H_2_O_2_, superoxide and lipid peroxide, all related to the pathophysiology of depression [185]. CA inhibits ROS-sensitive c-Jun N-terminal kinase 1/2 (JNK 1/2) and p38 MAPK signaling pathways through its antioxidant properties, thereby reducing NF-κB activation and inflammatory response [185,189,190,191].

#### 7.4.2. Mechanisms of Action of Coffee and Its Active Ingredients in Inflammation

Inflammation is considered an important pathological feature of depression and a risk factor for anxiety and SD [192,193,194,195]. Polymorphisms of cytokines and inflammatory mediators (IL-1β, TNF-α, C-C motif chemokine ligand-2 (CCL2) and C-reactive protein (CRP)) are associated with the severity of depression [196]. Proinflammatory cytokines and acute phase proteins are increased in MDD, suggesting that inflammation may be a key disease modifier promoting susceptibility to depression [197]. Peripheral and central inflammatory signals increase the release of proinflammatory cytokines, activate cyclooxygenase (COX-1/COX-2), and increase prostaglandin levels, further aggravating the inflammatory response. In addition, pro-inflammatory cytokines induce enhanced IDO and tryptophan 2,3-dioxygenase (TDO) activity, shifting tryptophan metabolism toward the KP, reducing 5-HT synthesis, and ultimately leading to 5-HT deficiency, exacerbating depression [198]. Inflammatory markers (such as CRP), neutrophils, and white blood cells) may reduce the volume of HPC and PFC regions. Affects CNS signal transduction, changes the levels of neurotransmitters (such as 5-HT, GABA), and aggravates depressive symptoms [199]. Inflammatory responses have been shown to be positively correlated with anxiety arousal, which may influence the overactivation of the amygdala in response to negative stimuli [200]. Under normal conditions, Na^+^/K^+^-ATPase (NKA) in microglia forms a complex with the purinergic receptor P2X7R, reducing P2X7R-mediated potass-ium ion (K^+^) efflux, thereby inhibiting microglia activation, reducing neuroinflammation, and achieving the effect of treating anxiety [201].

Caffeine and its primary metabolite paraxanthine have been demonstrated to inhibit LPS-stimulated TNF-α production in human whole blood. The inhibitory effect is mediated by the cAMP/PKA pathway [202]. CGA can significantly inhibit the release of proinflammatory mediators such as TNF-α in LPS-activated microglia. Its mechanism of action is mainly through blocking the phosphorylation and degradation of inhibitor of nuclear factor kappa-Bα (IκBα), thereby inhibiting the activtion and nuclear translocation of the NF-κB signaling pathway [203]. FA dimer and isoFA can have an anti-inflammatory effect similar to that of non-steroidal anti-inflammatory drugs by inhibiting the gene expression of COX-2 [204,205]. CA (10 μg/mL)can reduce the nitrite concentration in LPS-stimulated Raw 264.7 macrophages to regulate the concentration of inflammation-related substances and exert anti-inflammatory effects. CA showed inhibitory effects on LPS-induced NF-κB activity and the phosphorylation of inflammation-related signaling molecules JNK1/2 and p38 MAPK in the same cell line [189].

In conclusion, coffee and its main active ingredients may play a positive role in regulating mood through antioxidant and anti-inflammatory pathways. Future research can further focus on the specific role of coffee’s antioxidant and anti-inflammatory mechanisms in different human mood disorders, such as its mood regulation effect in chronic low-level inflammatory states.

### 7.5. Caffeine’s Regulatory Mechanism on the HPA Axis

HPA axis dysregulation is associated with MDD in adults and adolescents [206,207,208,209,210]. Its function is usually assessed by measuring the glucocorticoid cortisol or by the degree of suppression after dexamethasone stimulation [211,212,213]. The etiology of depression is believed to be related to the disorder of the classical stress response, including changes in GRs levels, decreased GRs sensitivity, and impaired negative feedback regulation function [214,215]. The dependence of anxiety-like behaviors on corticotropin releasing hormone (CRH) cells is more related to excitatory glutamatergic projections to a subset of peripheral neurons in the lateral hypothalamic cortex than to endocrine signals [216]. Patients with generalized anxiety, major depression, and other mood disorders often demonstrate HPA axis hyperactivity [217,218]. HPA axis dysregulation in patients with depression may be related to decreased 11β-HSD2 enzyme activity. Impaired function of this enzyme will lead to a decrease in the conversion of cortisol to cortisone, which will increase the level of active cortisol and continuously over activate the GRs, forming a vicious cycle of continued hyperactivity of the HPA axis [210]. Cortisol secretion shows a CR, with the lowest level at night, reaching a peak 30–60 min after waking up, and gradually falling during the day [219]. Poor sleep quality is associated with flatter diurnal cortisol levels, lower wakefulness values, or changes in the cortisol awakening response (CAR) [219,220,221,222].

Caffeine may activate the HPA axis by interacting with centrally located adenosine receptors in the hypothalamic afferent area, ultimately modulating corticotrophin releasing factor (CRF) and HPA axis activity. However, low doses of caffeine do not weaken the peak response of the HPA axis to stress [223]. Caffeine consumption during adolescence alters HPA axis function, leading to increased basal CORT levels during the circadian trough in early adulthood, reduced stress-induced ACTH and CORT release, and reduced adrenal sensitivity to ACTH [224]. Long-term caffeine use may interfere with the negative feedback regulation of the HPA axis by changing the expression of GRs/mineralocorticoid receptors (MRs) in key brain areas, leading to abnormal levels of stress hormones [64]. Regular caffeine intake may induce anxiety symptoms by affecting the functions of brain regions such as the PFC, hippocampus, and amygdala [64,139].

Therefore, future studies may focus on adolescents to explore the long-term effects of caffeine intake on HPA axis function (such as reduced adrenal sensitivity) in adulthood, and further reveal the relationship between this effect and emotion regulation.

### 7.6. Mechanisms of Coffee and Its Active Ingredients Influencing Depression, Anxiety, and SDs via the Microbiota-Gut–Brain Axis (MGB) Axis

#### 7.6.1. MGB Axis and Depression

The MGB axis connects the intestine to the CNS. Both the MGB axis and intestinal flora disorders are associated with the pathogenesis of a variety of neurological and psychiatric diseases, such as autism spectrum disorder, depression, anxiety, etc. [225,226]. The intestinal flora affects the functions of the immune system, HPA axis and other related systems through the gut–brain axis and the byproducts of metabolism, (such as Short-chain fatty acids (SCFAs)), thereby affecting emotions and behaviors. It also participates in the activation of the HPA axis to trigger immune responses and inflammation, and plays a key role in the development of depression [227]. SCFAs can cross the BBB and have shown anti-depressant and anti-anxiety effects in mouse experiments [228]. The microbial metabolite butyrate has been shown to improve the integrity of the intestinal barrier and BBB by upregulating the expression of hypoxia-inducible factor-1 (HIF-1). CMS combined with probiotic treatment can increase butyrate levels, thereby alleviating depressive behavior [229]. Probiotics can regulate the intestinal barrier function and indirectly stimulate inflammatory immune response, thereby significantly reducing serum cortisol levels and improving depression [230]. MGB axis dysregulation in early life is associated with growth retardation, decreased BDNF, enhanced HPA axis activation, impaired negative feedback of GRs, increased stress reactivity, abnormal brain development, impaired social interaction, anxiety-like manifestations, cognitive deficits and other abnormal behaviors, and may also induce metabolic, immune or psychological disorders inadulth-ood [231]. Rats subjected to chronic unpredictable mild stress (CUMS) and treated with gut microbiota transplantation (FMT) showed increased levels of hippocampal neurochemicals such as 5-HT, GABA, and BDNF, coupled with decreased inflammatory markers, leading to a reduction in depressive symptoms [232].

Coffee-induced changes in gut microbiota composition may translate into changes in effector molecules involved in gut–brain axis signaling, thereby influencing multiple neural and psychological processes [233]. Coffee is closely related to intestinal flora and CNS. It can improve the depressive behavior of rats, increase the levels of SOD and GPx in serum, reduce the levels of IL-6 and TNF-α in serum, and reverse intestinal dysbiosis [53]. Coffee can significantly improve the intestinal flora structure of rats fed a high-fat diet and significantly increase the level of SCFAs [234]. CGA pretreatment improved the depressive-like behaviors and serum biochemical levels (5-HT, DA, IL-6, and TNF-α) of rats with ACTH-induced depression, and also improved the reduction in fecal microbiota diversity in ACTH-treated rats [50].

#### 7.6.2. MGB Axis and Anxiety

Gut microbiota can regulate anxiety by influencing HPC and its related behaviors [192]. Anxiety in mice is accompanied by increased recruitment of hippocampal microglia (Iba1), monocytes (CD11b/CD45), and dendritic cells (CD11b/CD11c), as well as an increase in the number of brain neuron migration, NF-κB activation in the colon and brain, and increased expression of IL-β and TNF-α, leading to increased intestinal barrier permeability, that is, monocyte/macrophage activation triggers intestinal inflammation, and changes in intestinal flora induce anxiety through inflammatory response mechanisms [235]. When mice are exposed to microbial metabolites 4-ethylphenyl sulfate (4EPS), their oligodendrocytes become more immature and their myelin production decreases, which results in a thinning of the insulation around axons and causes anxiety [236].

Caffeine improves neuroinflammation and anxiety during sleep deprivation in rats by inhibiting microglial activation [237]. HPA axis reactivity is part of the bidirectional MGB axis. Caffeine can activate the HPA axis, which in turn stimulates the production of stress catecholamines (NE and AD). These substances are present at the host-microbiome interface such as the intestinal cavity and alveolar fluid, and trigger direct reactions in the microbiome, thereby indirectly affecting related states such as anxiety [238].

#### 7.6.3. Effects of Caffeine on Sleep via Intestinal Flora

CR and feeding behavior jointly influence diurnal variations in community composition and metabolic function in transgenic organisms [239]. The gut and brain communicate through the gut–brain axis, and the gut microbiota may influence areas of the brain that control CR [240]. Both sleep fragmentation and short sleep duration are associated with intestinal dysbiosis, which may be due to activation of the HPA axis. Insufficient sleep can alter the composition of the gut microbiota by increasing hunger and reducing physical activity, immune modulation or HPA axis activation, and subsequent intestinal barrier disruption. The gut microbiome can alter sleep through sleep-inducing LPS and muramyl peptides (MP) translocation, vagal afferent excitation in response to intestinal LPS, regulation of enterochromaffin cells (EC) 5-HT production, and modulation of inflammatory cytokines [241].

Caffeine-induced sleep restriction affects the composition of the gut microbiome and fecal metabolites in a mouse model [242]. Severe sleep deprivation has been shown to lead to ROS accumulation in the gut, and taking antioxidants such as melanoidins found in coffee can prevent this effect [243]. Caffeine can modulate lipid metabolism pathways in the gut microbiota to reduce metabolic disturbances and lipid accumulation induced by sleep restriction [242]. The molecular mechanism of coffee and its active ingredients on depression, anxiety and SD is shown below (Figure 3).

In summary, coffee can relieve depression and anxiety symptoms by changing the composition of intestinal flora and then affecting brain neural activity. Future research can further explore: (1) personalized coffee intake strategies based on individual gut microbiota characteristics. (2) long-term effects of coffee intervention on sleep and mood in special populations (patients with metabolic syndrome). Exploring the effects of coffee processing methods (such as roasting degree) on intestinal flora metabolites can provide a basis for the development of related coffee products.

## 8. Conclusions and Outlook

This review examined studies on coffee and its bioactive compounds (e.g., caffeine, polyphenols) in relieving depression, anxiety, and SDs, covering animal models, clinical trials, and epidemiological analyses. According to current research results, coffee may affect mood through multiple mechanisms. And coffee has a dual effect on mood: moderate intake can enhance cognitive function and improve mood, but excessive intake may be related to anxiety, insomnia, PTSD, etc.

In addition, the exact mechanisms of coffee’s effects on mental health remain unclear. Future research should focus on the following: (1) There is still a need to further explore the mechanisms by which coffee and its various active ingredients affect brain function and mood. There should be more in-depth research on how coffee and its active ingredients (including metabolites) regulate specific intestinal flora to produce neuroactive substances, thereby exerting a protective effect on brain function. (2) Based on the regulatory effect of caffeine on CR, explore its potential therapeutic strategy for intervening in rhythmic disorders associated with metabolic diseases. (3) Study the effects of different coffee varieties, roasting, and brewing methods on coffee’s bioactive components and their effects on mood, explore the appropriate dosage for drinking coffee, and provide a basis for the development of functional foods containing coffee’s active ingredients.

## 9. Strengths and Limitations

This study has some strengths as follows: (1) This study explores the association and impact between coffee and its active ingredients and depression, anxiety and SDs, providing valuable guidance for subsequent research. (2) This study systematically reviews the research results on coffee and its ingredients in depression, anxiety and SDs, covering animal experiments, clinical trials and epidemiological studies, and the content is detailed and comprehensive. (3) The description of the mechanism of action of coffee and its components is relatively detailed, including adenosine receptor regulation, regulation of neurotransmitters, antioxidant and anti-inflammatory effects, and so on.

However, there are some limitations: (1) The article discusses the mechanism of action of coffee, but there is still a lack of clear recommendations on the practical application of coffee, such as recommended intake or suitability for specific populations. (2) The article cited a large number of studies, but lacked in-depth evaluation of the limitations and reliability of the methodologies of these studies, such as the sample size of the experimental design and whether the control group setting was adequate, resulting in insufficient depth of critical analysis in the study.

## Figures and Tables

**Table 1:** Animal experimental studies on the effects of coffee and its active ingredients on depression, anxiety, SDs.

Mental Diseases	Coffee and Its Active Ingredients	Animal Models	Dose and Duration	Behavioral Manifestations	Results and Mechanisms	References
Depression	Caffeinated and decaffeinated coffee (Coffee Extracts)	PDD inbred male Wistar rats	200 mg/kg (p.o.), 7 d	Sports activities↑(OFT) Sucrose consumption↑ (SPT) Immobility time↓ (FST) Immobility time↓ (TST)	IL-6↓ TNF-α↓ SOD↑ GPx↑ Akkermansia (Verrucomicrobia)↑ Phylum Bacteroidetes (Bacteroidetes)↓	[53]
	Caffeinated coffee (Coffee extracts)	Male C57BL/6J mice	140 mg/kg (p.o.), 14 d	Sports activities↑ (OFT) Immobility time↓ (FST) Immobility time↓ (TST)	IDO↑ PGE2↓ Neopterin/biopterin ratio↑ UA/3-HK, UA/KYN↑	[55]
	Caffeine (Pure ingredients)	Male C57BL/6 mice (10–12 weeks old)	6 g/L/week (p.o.), 3 weeks	Immobility time↓ (FST) Immobility time↓ (TST) Pleasure deficit-like behavior↓ (SPT) memory impairment↓	synaptic marker↓ hippocampal density of syntaxin↓ amplitude of hippocampal LTP↓	[56]
	Caffeine (Pure ingredients)	Male C57BL/6J mice (Chronic MildStress)	0.5 mg/mL (p.o.), 6 weeks	Immobility time↓ (FST) Immobility time↓ (TST)	KYN↓ KYNA↑ KAT↑ PGC-1α↑	[58]
	Caffeine (Pure ingredients)	Kunming male mice (Chronic Water Immression Restraint Stress)	10, 20 mg/kg (p.o.), 4 weeks	Immobility time↓ (FST) Immobility time↓ (TST)	5-HT↑ BrdU+/DCX+ cells↑ TNF-α↓ IL-1β↓	[57]
	Caffeine (Pure ingredients)	Sprague-Dawley rats	0.25 g/L (p.o.), 14 d	Upright behavior of male rats↓ Immobility time of male rats↓(FST) Struggling behavior of female rats↑	Changes in 5-HT levels Regulation of NE	[59]
Depression	Caffeine (Pure ingredients)	Swiss albino CD1 mice (Helpless Mice)	0.3 g/L (p.o.), 4 weeks	Immobility time↑ (FST) Immobility time↑ (TST) Time spent in the open arms↓ Length of stay in the central area↓ Memory recovery↑	density of synaptophysin, SNAP-25↓ Restored loss of hippocampal synaptic markers	[62]
	Caffeine (Pure ingredients)	Male C57BL/6J mice	10 mg/kg (p.o.), 14 d	Immobility time↓ (FST) Immobility time↓ (TST)	TNF-α↓ IL-6↓ IL-1β↓ IFN-γ↓ MDA↓ BDNF↑	[61]
	SY-2476 (Caffeine derivative, pure ingredients)	Wistar rats	10, 30 mg/kg (p.o.), 21 d	Sucrose consumption↑ (SPT) Immobility time↓ (FST)	Serum cortisol levels↓ MDA↓ SOD↑ GSH↑ A_1_R expression↑ A_2A_Rexpression↓	[60]
	caffeine (Pure ingredients)	Male Wistar rats (Stress Re-stress)	10, 20, 30 mg/kg (i.p.), 24 d	Immobility time (20, 30 mg/kg)↑ (FST)	Plasma CORT levels, 5-HT levels, GR and MR levels were not changed	[64]
	CGA (Pure ingredients)	Male Wistar rats	500 mg kg^−1^ (p.o.), 14 d	Sucrose consumption↑ (SPT) Sports activities↑ (OFT) Immobility time↓ (FST) Immobility time↓ (TST)	IL-6 and TNF-α↓ Microbiome diversity↑ Serum pro-inflammatory cytokine↓ Serum monoamine neurotransmitter↑ IL-8↓	[50]
	FA (Pure ingredients)	Male Swiss mice	0.01, 0.1, 1, 10 mg/kg (p.o.), 1 h	Immobility time↓ (FST) Immobility time↓ (TST)	5-HT↑ SNRIs↑	[66]
	CA (Pure ingredients)	Male ICR mice and ddY mice	4 mg/kg (i.p.), 1 d	Immobility time↓ (FST)	Indirect regulation of the ADRA1A system	[67]
Anxiety	Caffeine (Pure ingredients)	Male Sprague Dawley rats	3 mg/kg (i.p.), 10 min (acute stress) or 14 d (Chronic variable stress)	Head Down Frequency↑	Cognitive Differences Score↑ MDA↓ SOD (acute)↑ GSH (acute)↑	[68]
	Caffeine (Pure ingredients)	Male C57BL/6 mice	5 mg/kg (i.p.), 24 d	Open arm dwell time (short-term effect) ↑(EPM)	MDA↓NO↓	[69]
	Caffeine (Pure ingredients)	Male Wistar rats (Stress Re-stress)	10, 20, 30 mg/kg (i.p.), 24 d	Open arm dwell time (20, 30 mg/kg) ↓(EPM)	Plasma CORT levels, 5-HT levels, GR and MR levels were not changed	[64]
	CGA, Caffeine (Pure ingredients) Coffee (Coffee Extracts)	Adult male Wistar rats	CGA: 5 mg/kg (p.o.), Caffeine: 15 mg/kg (p.o.), Coffee: 0.5 g/kg (p.o.), 29 d	Step-down latencies↑ (IAT)	TBARS Level↓ AChE activity↓ Nerve damage↓	[70]
	ICCA (Coffee Extracts)	Male ICR mice	20, 40 mg/kg (p.o.), 3 months	Open arm dwell time and access frequency↑ (EPM) Light box dwell time↑ (LDB)	SH-SY5Y cells, B16 cells, H9C2 cells↓ OS↓ TNF-α and IL-6↓ Nrf2↑	[71]
	CGA (Coffee Extracts)	Swiss albino male mice	20 mg/kg (i.p.), 1 h	Light box dwell time↑ (LDB) Time spent in the open arms and frequency of entering the open arms↑ (EPM) Feeding behavior, exercise ability↑ (FET)	Benzodiazepine receptors↑ OS↓	[72]
Anxiety	CGA SYG (Pure ingredients)	Male Sprague Dawley rats	CGA: 40 mg/kg (p.o.), 7 d SYG: 32 mg/kg (p.o.), 7 d	Time spent in open arms↑ (EPM)	Hippocampal BDNF signaling↑ PNS activity↑	[73]
	CGA (Pure ingredients)	Male Wistar rats	50 mg/kg (p.o.), 8 weeks	Average time to reach the underwater platform↓ The average swimming distance↓ (MWM) Light box dwell time↑ (LDB) Number of marbles buried↓ (MBT)	MDA↓ BDNF↑ IL-1β↓ IL-2↓ TNF-α↓ INF-γ↓	[74]
	FA (Pure ingredients)	Male C57BL/6J mice	20 mg/kg (p.o.)	Sports activities↑ (OFT) Immobility time↓ (FST) Immobility time↓ (TST) Sucrose consumption↑ (SPT) Open arm dwell time and access frequency↑ (EPM)	Firmicutes/Bacteroidetes ratio↑ 5-HTP↑	[75]
SDs	Caffeine (Pure ingredients)	Male A1R knockout mice, A2AR knockout mice, wild-type C57BL/6 mice	2.5, 5, 10, 15 mg/kg (i.p.), 3 h	Wake up time (5, 10, 15 mg/kg) ↑ NREM and REM↓	A2AR-mediated	[76]
	Caffeine (Pure ingredients)	Wild-type C57Bl6 mice	0.3, 0.6, 0.9 and 1.2 mg/mL (p.o.), 21 d	Resting phase wakefulness block↓ Quiescent phase↓ Active period↑ REM↓ Sleep delay↑	Fluctuation of adenosine receptor antagonism	[77]
SDs	CGA, CA and caffeine (Pure ingredients)	Male Wistar rats	CGA: 50, 100, 200, 500 mg/kg CA:20, 50, 100, 200 mg/kg; caffeine: 1, 2, 5, 10 mg/kg, (p.o.), 35 d	Sleep latency↑ Wake up time↑ NREM↓	Activation of the α1-AR system	[78]
	Caffeine (Pure ingredients)	C57BL/6 mice	0.13, 0.26 g/kg (i.p.), 12 min	Level of spontaneous activity↓	sympathetic nervous system activity↑	[79]
well-being	Caffeinated and decaffeinated coffee (Coffee Extracts)	C57BL/6J mice	1 g/L (p.o.), 3 weeks	Caffeinated coffee: Males: Open arm dwell time ↓(EPM) Upright behavior and climbing time ↑ Grooming time and social time↑ Number of buried glass spheres↑ Females: Selfcare ↑	Caffeinated: A_1_R Increased in striatum (Males) A_1_R increased in the ventral hippocampus (Females) Males and Females: MDA↓ GSH↑	[80]

Notes: “p.o.”: Per oral, “i.p.”: Intraperitoneal injection, “↓” represents downregulation, “↑” represents upregulation.

**Table 2:** Clinical studies on the effects of coffee and its active ingredients on depression, anxiety and SDs.

Mental Diseases	Types of Studies	Coffee or Its Active Ingredients	Participants	Dose and Duration	Results and Conclusions	References
Depression	Randomized, double-blind, sham-controlled trial	caffeine	N = 40 (age: 18–59 years)	Self-reported number of cups of coffee and energy drinks consumed, 10–15 d	Antidepressant effect of dorsomedial iTBS↑ Depression↓	[81]
	Randomized, double-blind, crossover study	Caffeinated and decaffeinated coffee	N = 77 (mean age: 20.38 ± 1.28 years)	150 mg (caffeinated coffee), 1 d	Post-treatment self-report questionnaire scores↑ SBP and DBP↑ Depression↓	[82]
	Randomized, double-blind, controlled trial	Caffeinated and decaffeinated coffee	N = 88 (age: 18–47 years)	75 mg/d (caffeinated coffee), 1 d	Stroop task accuracy↑ Card arrangement rewards responsiveness and sorting speed↑ Depressed mood↓	[83]
Anxiety	Randomized, double-blind, controlled study	caffeine	N = 56 (mean age: 69.2 years, female)	200 mg/d, 400 mg/d, 7 d	Anxiety symptoms↓ Low or moderate caffeine may reduce anxiety	[86]
	Randomized, controlled, crossover study	caffeine	N = 16 (Caffeine withdrawal subjects)	100 mg/d, 1 d	SBP and DBP↑ Anxiety symptoms↓	[87]
	Prospective, open-label trial	Feru-guard	N = 20 (patients with frontotemporal lobar degeneration or dementia with Lewy bodies)	3 g/d (Feru-guard), 4 weeks	Neuropsychiatric Scale Total Score↓ Dementia, anxiety symptoms↓ May be effective for treating frontotemporal lobar degeneration or Lewy body	[88]
SDs	Randomized, double-blind, controlled, crossover study	CGA	N = 16 (age: 30–54 years, Healthy male)	300 mg/d, 14 d	Awakening fatigue↓ Sleep quality↑ Sleep quality↑	[89]
	Randomized, double-blind, controlled, crossover study	CGA	N = 9 (mean age: 25.7 years, healthy adults)	600 mg/d, 5 d	Sleep latency↓ Parasympathetic activity↑ No adverse effect on sleep quality	[90]
	Randomized, double-blind, controlled, crossover study	caffeine	N = 5 (mean age: 24.0 ± 2.8 years)	2.9 mg/kg, 49 d	The CR is delayed	[91]
	Randomized, double-blind, controlled, crossover study	caffeine	N = 15 (mean age: 23.7 ± 8.2 years)	6 mg/kg, 1 d	sleep quality↓	[92]
	Randomized, double-blind, controlled, crossover study	caffeine	N = 20 (mean age: 26.4 ± 4 years)	450 mg/d, 9 d	EEG power density↓ Nocturnal sleep structure or subjective sleep quality is not greatly affected	[93]

Notes: “↓” represents downregulation, “↑” represents upregulation.

**Table 3:** Epidemiological studies on the effects of coffee and its active ingredients on depression and anxiety.

Mental Diseases	Participants	Coffee or Its Active Ingredients	Conclusions	References
Depression	N = 9576 (age: ≥19 years)	Coffee and Caffeine	Regular coffee or caffeine consumption is associated with lower self-reported lifetime depression prevalence in adults	[94]
	N = 50,739 (mean age: 63, female)	Caffeinated coffee	Depression risk decreases with increasing caffeinated coffee intake, with 2–3 cups being most effective	[95]
	N = 821 (Postpartum women)	Caffeinated coffee	Moderate coffee consumption (1–2 cups per day) may reduce the risk of PDD, while higher consumption (3 cups per day or more) has no significant effect	[97]
	N = 80,497	Black Coffee	Moderate drinking of black coffee (4–6 times/week) may have a certain preventive effect on depression	[98]
Anxiety	N = 146,566 (from UK Biobank)	Coffee	Moderate coffee consumption (1–4 cups/day) showed a trend of reducing the risk of anxiety	[52]
	188,355 (age: 37–73, from UK Biobank)	Coffee	For individuals aged ≥ 60 years, higher coffee intake was negatively associated with anxiety disorders	[99]

## Data Availability

No new data were created or analyzed in this study. Data sharing is not applicable to this article.

## Abbreviations

The following abbreviations are used in this manuscript:

SDs	Sleep disorders
MDD	Major depressive disorder
TCAs	Tricyclic drugs
5-HT	Serotonin
SSRIs	Selective Serotonin Reuptake Inhibitor(s)
NE	Norepinephrine
SNRI	Serotonin–norepinephrine reuptake inhibitors
DXT	Duloxetine
DVS	Desvenlafaxine succinate
GAD	Generalized Anxiety Disorder
SAD	Social Anxiety Disorder
PTSD	Post-traumatic stress disorder
OCD	Obsessive–compulsive disorder
PSD	Post-stroke depression
CGA	Chlorogenic acid
FA	Ferulic acid
CA	Caffeic acid
DA	Dopamine
PFC	Prefrontal Cortex
A1R	A1 receptors
A2AR	A2A receptors
ACTH	adrenocorticotropic hormone
DMN	Default mode network
MAO-A	Monoamine oxidase-A
DSM-IV	Diagnostic and Statistical Manual of Mental Disorders-IV
SPT	Sugar preference test
FST	Forced swim test
TST	Tail suspension test
OFT	Open field test
IL-6	Interleukin-6
TNF-α	Tumor necrosis factor-α
SOD	Superoxide dismutase
GPx	Glutathione peroxidase
PDD	Postpartum Depression
IDO	Indoleamine 2, 3-dioxygenase
LPS	Lipopolysaccharide
UA	Uric acid
3-HK	3-Hydroxykynurenine
KYN	Kynurenine
KP	Kynurenine pathway
CNS	Central Nervous System
CUS	Chronic unpredictable stress
LTP	Long-term potentiation
CORT	Corticosterone
MAPK	Mitogen-activated protein kinase
ERK	Extracellular regulated protein kinases
NF-κB	Nuclear Factor kappa-light-chain-enhancer of activated B cells
CWIRS	Chronic water immersion restraint stress
CMS	Chronic Mild Stress
PGC-1α	Peroxisome proliferators-activated receptor γ coactivator lα
KAT	Kynurenine aminotransferase
KYNA	Kynurenic acid
SRS	Stress-induced stress
PGE2	Prostaglandin E2
BrdU	Bromodeoxyuridine
DCX	Doublecortin
IL-1β	Interleukin-1β
SNAP-25	Synaptosomal-Associated Protein 25
IFN-γ	Interferon-gamma
MDA	Malondialdehyde
BDNF	Brain-derived neurotrophic factor
GSH	Glutathione
GR	Glucocorticoid receptor
ADRA1A	α1Aadrenoreceptor
TBARS	Thiobarbituric acid reactive substances
AChE	Acetylcholinesterase
OS	Oxidative stress
Nrf2	Nuclear factor-erythroid 2 related factor 2
EPM	Elevated plus maze test
LDB	Light/dark box test
IAT	Inhibitory avoidance test
FET	Free exploration test
PNS	Peripheral nervous system
MWM	Morris water maze
MBT	Marble burying test
5-HTP	5-Hydroxytryptophan
REM	Rapid eye movement sleep
NREM	Non-rapid eye movement sleep
Per1/Per2	Period Circadian Regulator 1/2
α1-AR	α1-adrenergic receptor
iTBS	Intermittent burst theta stimulation
dmPFC	Dorsomedial prefrontal cortex
SBP	Systolic Blood Pressure
DBP	Diastolic Blood Pressure
CR	Circadian Rhythm
EEG	Electroencephalogram
ICCA	Isochlorogenic acid
SYG	Syringaresinol-di-O-glucoside
GSK-3β	Glycogen synthase kinase-3β
OAB	Overactive bladder
CSF	Cerebrospinal fluid
SCN	Suprachiasmatic nucleus
CREB	Cyclic-AMP response binding protein
OXRs	Orexin receptor antagonists
NMDA	N-methyl-d-aspartate
PKC	Protein kinase C
cAMP	Cyclic adenosine monophosphate
LS	Lateral septal
SNP	Single nucleotide polymorphism
PAG	Periaqueductal gray
mPFC	Medial prefrontal cortex
AANAT	Aryl alkylamine N-acetyltransferase
CRTC1	CREB-regulated transcription coactivator 1
CRE	cAMP response element
PI3K	Phosphatidylinositol 3-kinase
mTOR	Mechanistic target of rapamycin
TrkB	Tropomyosin-related kinase B
PLC-γ	Phospholipase C-γ
AMPAR	α-amino-3-hydroxy-5-methyl-4-isoxazolepropionic acid receptor
NAc	Nucleus accumbens
IRS2	Insulin receptor substrate 2
CAFLTP	Caffeine-induced LTP
P-CaMKII	Phosphorylated calcium calmodulin kinase I
GABA	Gamma-aminobutyric acid
CCK	Cholecystokinin
AvBNST	Anteroventral nucleus of the bed nucleus of the stria terminalis
VTA	Ventral tegmental area
DRN	Dorsal raphe nucleus
BLA	Basal lateral amygdala
ROS	Reactive oxygen species
HPA axis	hypothalamic–pituitary–adrenal axis
GCs	Glucocorticoids
H_2_O_2_	Hydrogen peroxide
·OH	Hydroxyl radicals
ROO·	Peroxyl radicals
1O2	Singlet oxygen
JNK1/2	c-Jun N-terminal kinase 1/2
CCL2	C-C motif chemokine ligand-2
CRP	C-reactive protein
TDO	Tryptophan 2,3-dioxygenase
NKA	Na+/K+-ATPase
IκBα	Inhibitor of nuclear factor kappa-Bα
CRH	Corticotropin releasing hormone
CAR	Cortisol awakening response
CRF	Corticotrophin releasing factor
MRs	Mineralocorticoid receptors
MGB axis	Microbiota-Gut–Brain axis
SCFAs	Short-chain fatty acids
HIF-1	Hypoxia-inducible factor-1
CUMS	Chronic unpredictable mild stress
FMT	Fecal microbiota transplantation
4EPS	4-Ethylphenyl sulfate
MP	Muramyl peptides
EC	Enterochromaffin cells
